# Chronotype and psychopathology: insights from a genetically informative design

**DOI:** 10.1093/sleep/zsaf231

**Published:** 2025-09-10

**Authors:** Juan J Madrid-Valero, Juan R Ordoñana, Thalia C Eley, Alice M Gregory

**Affiliations:** Department of Human Anatomy and Psychobiology, University of Murcia, Murcia, Spain; Murcia Institute for Biomedical Research (IMIB-Arrixaca), Murcia, Spain; Department of Human Anatomy and Psychobiology, University of Murcia, Murcia, Spain; Murcia Institute for Biomedical Research (IMIB-Arrixaca), Murcia, Spain; Social, Genetic and Developmental Psychiatry Centre, Institute of Psychiatry, Psychology and Neuroscience, King's College London, London, United Kingdom; National Institute for Health and Care Research (NIHR) Maudsley Biomedical Research Centre, South London and Maudsley NHS Foundation Trust, London, United Kingdom; Department of Psychology, Royal Holloway, University of London, London, United Kingdom

**Keywords:** chronotype, genetics, p-factor, polygenic scores, psychopathology, twins

## Abstract

**Study objectives:**

Chronotype has been linked to a wide variety of psychiatric conditions. In particular, evening chronotype could be a transdiagnostic risk factor for different mental health difficulties. In this study, we examine how chronotype relates to psychopathology and whether it can be conceptualized as a part of the global construct of psychopathology (p-factor) by studying the genetic and environmental overlap between these variables. We utilize data from a genetically informative design to study: (1) the association between chronotype and psychopathology; (2) the genetic and environmental overlap between chronotype and psychopathology; and (3) the predictive value of polygenic score (PGS) for chronotype for psychopathology.

**Methods:**

Chronotype was measured using an abbreviated version of the Munich Chronotype Questionnaire. Measures of psychopathology included: depression, anxiety, alcohol use, and psychotic experiences among others. We used different psychopathology and chronotype-related polygenic scores. Association between chronotype and psychopathology were examined with three approaches: (1) phenotypic associations; (2) genetic and environmental associations using the twin design; and (3) genetic associations using PGS.

**Results:**

There were small, though largely significant, associations between chronotype and psychopathology with significant genetic and environmental overlap. Chronotype PGS significantly predicted a very small proportion of the variance for some measures of psychopathology (e.g. symptoms of attention deficit hyperactivity disorder). However, overall, our results also suggest that the majority of genetic/environmental influences (96 per cent) on chronotype do not overlap with those on the psychopathology factor.

**Conclusions:**

Results from this study highlight existence of significant associations between chronotype and certain psychopathology traits. However, the very small associations do not support the idea that chronotype is a core element of the general “p-factor.”

## Teaser

**Statement of Significance** Chronotype (and primarily late chronotype) has been linked to a wide variety of psychiatric conditions. This study deepens understanding of the association between chronotype and psychopathology, by comprehensively exploring this association using a genetically informative design. Our results reveal small but largely significant associations between chronotype and mental health variables and provide new insights into the etiology of these associations. This report considers whether late chronotype, which many consider to be a transdiagnostic risk factor for psychopathology, could be integrated in the global factor of psychopathology. Overall, our results suggest that while there are significant associations between chronotype and psychopathology traits—these associations are small and chronotype is largely influenced by genetic/environmental influences distinct from those on the psychopathology factor (more than 96 per cent) and should not be considered as a core feature of this construct.

## Graphical Abstract

**Graphical Abstract:**
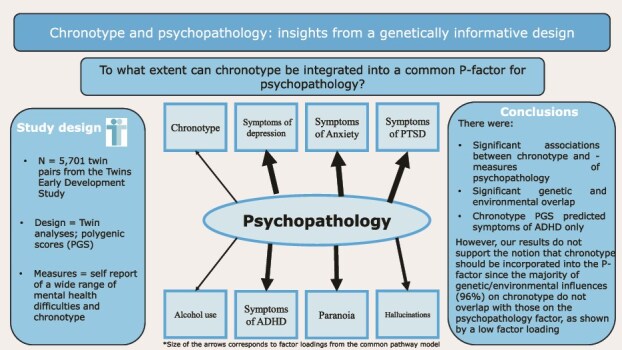

## Introduction

Chronotype captures the tendency to behave in a certain way at certain times of day, due to underlying circadian rhythms. For example, some people have a tendency and preference to go to sleep earlier than others; and some may have greatest alertness early in the day, with others reporting greater alertness later in the day [1]. Chronotype has been linked to a wide variety of psychiatric conditions. Specifically, “eveningness” (a tendency and preference to be active later in the 24-h day) has been associated with psychopathology [1, 2]. Indeed, it has been suggested that an evening chronotype could be a transdiagnostic risk factor for different mental health disorders, with longitudinal studies suggesting that evening chronotype precedes psychopathology [2, 3]. Numerous studies have examined the association between chronotype and specific types of psychopathology. For example, one meta-analysis found that an evening orientation was associated with more severe symptoms of depression and a greater likelihood of suffering from this disorder [4]. Nonetheless, research is mixed as it has also been reported that morningness is associated with higher rates of depression [5]. Anxiety has also been linked to eveningness in adults (morning chronotype could be protective for anxiety), although results in adolescents and young adolescents are sometimes contradictory and some studies have not found any association between chronotype and anxiety at all [6, 7]. Chronotype has also been associated with externalizing behaviors—with eveningness associated with increased levels of aggression and antisocial behavior [8], attention deficit hyperactivity disorder (ADHD) [9], and alcohol problems, among other mental health difficulties [10]. Finally, some studies have looked at associations between chronotype and psychopathology more broadly. For example, one study of more than 160 000 university students found that being an “evening type” was associated with increased mental health difficulties and sleep problems for both men and women [11].

Individual differences in chronotype are substantially (40–52 per cent) influenced by genetic factors [12–15]. Previous research has also found significant genetic correlations between chronotype and mental health variables such as depression [12] and externalizing behaviors [16]. While chronotype is associated with a wide range of traits, the mechanisms underlying these associations are unclear. Given genetic influences on both psychopathology [17] and sleep phenotypes including chronotype [18], the possibility of genetic influences on these association should be explored further [2] to understand more about why these variables co-occur. Despite these important contributions, there are no studies that have investigated genetic and environmental influences on the association between chronotype and a wide variety of psychopathological traits—which is particularly significant given that chronotype has been proposed as transdiagnostic.

Previous research has revealed that a common p-factor (i.e. a general factor underlying different psychopathologies) is substantially heritable (50–60 per cent) [17]. Given that chronotype is considered a transdiagnostic factor for psychopathology and that it is biologically plausible for sleep disturbance to be mechanistically transdiagnostic [19], here we examine how chronotype relates to psychopathology and whether it can be integrated in the p-factor. Our objectives are thus to utilize data from a genetically informative design to study: (1) the association between chronotype and psychopathology; (2) the genetic and environmental overlap between chronotype and psychopathology; and (3) the value of PGS for chronotype to predict psychopathology.

## Materials and Methods

### Participants

The sample initially comprised 11 402 twins (5701 twin pairs) from the Twins Early Development Study (TEDS). We excluded 249 twin pairs due to medical reasons (twins with serious medical conditions which affect their ability to take part in the study), perinatal outliers (e.g. very low birth weight or long period of special care after birth), or unknown zygosity. TEDS is a community-based, longitudinal study of twins born in England and Wales between 1994 and 1996 [20–22]. This sample has been shown to be reasonably representative of the general population [23]. Zygosity was assessed using either DNA testing or a questionnaire, which has shown an accuracy of over 95 per cent [24]. Ethical approval for TEDS was provided by the King’s College London ethics committee (ref: PNM/09/10-104). Written informed consent was obtained prior to each wave of data collection from parents and from twins themselves from age 16 years onward. This work focuses on data collected at age 26 years, the first wave at which chronotype has been assessed. Information about descriptive statistics can be found in Table 1. The sample was 59.5 per cent female and comprised both monozygotic (MZ; 34.8 per cent) and dizygotic twins (DZ; 65.2 per cent).

**Table 1:** Descriptive Statistics

Variable	**Male**	**Female**	**MZ**	**DZ**	**Total sample**
Total *N* (%)	4419 (40.5)	6485 (59.5)	3800 (34.8)	7104 (65.2)	10 904 (100)
Mean age (SD)	26.47 (0.91)	26.4 (0.93)	26.46 (0.92)	26.4 (0.92)	26.42 (0.92)
Mean chronotype (*N* = 7458)	04:00:32	03:36:33	03:44:39	03:44:48	03:44:44
Mean symptoms of depression (SD) (*N* = 8238)	5.84 (5.68)	7.41 (6.51)	6.8 (6.31)	6.9 (6.26)	6.86 (6.28)
Mean symptoms of anxiety (SD) (*N* = 7959)	6.14 (6.57)	8.52 (7.5)	7.59 (7.22)	7.77 (7.31)	7.7 (7.28)
Mean alcohol use (SD) (*N* = 7152)	7.46 (5.01)	6.06 (4.52)	6.47 (4.7)	6.58 (4.77)	6.54 (4.75)
Mean callous unemotional traits (SD) (*N* = 7564)	4.74 (2.94)	3.26 (2.52)	3.74 (2.77)	3.78 (2.76)	3.77 (2.76)
Mean symptoms of ADHD (SD) (*N* = 7573)	6.8 (6.16)	6.53 (6.19)	6.51 (6.1)	6.69 (6.23)	6.62 (6.18)
Mean autistic traits (SD) (*N* = 7578)	2.04 (2.54)	2.33 (2.79)	2.17 (2.7)	2.27 (2.71)	2.23 (2.71)
Mean symptoms of PTSD (SD) (*N* = 7688)	4.56 (5.01)	5.93 (5.87)	5.37 (5.56)	5.51 (5.67)	5.46 (5.63)
Mean specific psychotic experiences: paranoia (SD) (*N* = 7570)	6.38 (10.6)	7.54 (11.68)	7.03 (11.04)	7.2 (11.5)	7.14 (11.33)
Mean specific psychotic experiences: hallucinations (SD) (*N* = 7568)	1.09 (3.46)	1.26 (3.75)	1.15 (3.6)	1.23 (3.69)	1.2 (3.65)
Total *N* eating disorder (%) (*N* = 7521)	36 (1.4)	385 (7.8)	158 (5.6)	263 (5.6)	421 (5.6)

ADHD: attention deficit hyperactivity disorder; PTSD: post-traumatic stress disorder; SD: standard deviation.

### Measures

Chronotype was measured using a reduced version of the Munich Chronotype Questionnaire (MCTQ) [25]. This questionnaire assesses the timing of sleep within a typical 24-h day and provides a measure that allow us to estimate individual internal time. Given that we did not include all the MCTQ items in our survey, we used the scoring from the ultra-short version of this measure [26]. This coding has shown good test–retest reliability and has correlated significantly with phase markers from actimetry and melatonin [26]. We did not remove participants who stated that they used an alarm clock during free days (around 20 per cent) since that would mean losing information not only for that participant but also for the twin pair. However, we performed sensitivity analyses removing those who reported using an alarm to wake during free days in order to check the impact of this factor and found that the results were substantively identical for both the associations between chronotype and measures of psychopathology as well as for the genetic and environmental influences on chronotype (see results section and Supplementary Material).

We also used a wide variety of measures of psychopathology (selected based on previous publications addressing the p-factor in the TEDS sample [17]). Symptoms of depression were measured using the Short Mood and Feelings Questionnaire (SMFQ). The SMFQ consists of 13 items with responses for each from 0 (not true) to 2 (true) and these scores can yield a total score ranging from 0 to 26. These questions assess key symptoms of depression and focus on the previous 2 weeks [27].

Symptoms of anxiety were measured by means of the General Anxiety Dimensional measure (GAD-10). This scale has 10 items ranging from 0 to 4 (range of the scale 0–40) [28].

Alcohol use was measured using an adapted version from the Alcohol Use Disorders Identification Test. This scale has 10 items coded from 0 to 4 (range of the scale 0–40) [29].

Callus unemotional traits were measured using a reduced version of the Inventory of Callous Unemotional traits (reduced from 24 to 7 items (coded from 0 to 3); range of the questionnaire 0–21) [30, 31].

Symptoms of ADHD were measured using the Conners scale which has 11 items each coded from 0 to 3 (range of the questionnaire 0–33) [32].

Autistic traits were assessed using a reduced version of the Ritvo Autism and Asperger Diagnostic Scale retaining 6 items (coded from 0 to 3) out of 14 (3 socio-communicative and 3 non-social items; range of the questionnaire 0–18) [33].

Symptoms of post-traumatic stress disorder were assessed using an abbreviated version from the Post-Traumatic Stress Disorder Checklist with 6 items ranging from 0 to 4 (range of the scale 0–24) [34].

Paranoia and hallucinations were measured using subscales of the Specific Psychotic Experiences Questionnaire. Paranoia was assessed by 15 (ranging from 0 to 75) items and hallucinations by 9 items (ranging from 0 to 45), both rated on a 6-point scale (0–5) [35].

Eating disorders were assessed using criteria from the Diagnostic and Statistical Manual of Mental Disorders (DSM-5). This variable was then coded as follows: 0 = “not diagnosed,” 1 = “diagnosed without subtype,” 2 = “diagnosed with restricting subtype,” 3 = “diagnosed with purging/binge-eating subtype.” For the analysis, this variable was dichotomized (0 = “not diagnosed”; 1 = “any diagnosis”).

### Polygenic scores

PGS for chronotype and 18 measures of psychopathology and health were used (Table S1). All these PGS were previously constructed in TEDS. Where TEDS had formed part of the discovery GWAS sample, PGS were created from summary statistics generated without the contribution of the TEDS cohort. PGS were calculated using LDpred which re-weights the variant effect sizes using a prior on their effect size (based on the heritability and assumed fraction of causal markers that influence the trait), adjusting for the linkage disequilibrium in the sample [36]. Details about genotyping can be found elsewhere (https://www.teds.ac.uk/datadictionary/studies/dna.htm). PGS were adjusted for the first 10 principal components, chip, and plate using the regression method and were *z*-standardized (mean = 0, SD = 1) to avoid potential effects of population stratification and genotyping. When several PGS where available for the same trait, we selected the most updated one.

### Statistical analysis

This study was preregistered on the Centre for Open Science Website (https://osf.io/xua56). Analyses of this study are divided into three main parts to study the association between chronotype and psychopathology: (1) phenotypic associations, (2) genetic and environmental associations using twin designs, and (3) genetic associations using PGS. We fitted a series of regression models to test whether PGS for chronotype predicts different aspects of psychopathology; and whether psychopathology PGS predicts chronotype. These analyses allowed us to test whether a genetic predisposition to chronotype is related to higher levels of psychopathology (and vice versa). Using two distinct methods to address our questions allows for the triangulation of findings, strengthening confidence in the results.

#### Phenotypic association

Phenotypic associations between chronotype and measures of psychopathology were tested using Pearson’s correlations for all the continuous variables and a point-biserial correlation for the eating disorder variable. These correlations were calculated using one randomly selected twin from each twin pair to ensure the independence of observations. To avoid decomposing very small associations, only variables that showed a correlation above 0.1 were included in subsequent twin models.

#### Twin models

The classical twin design allows us to disentangle the role of genetic and environmental influences on one trait or behavior [37]. The logic can be summarized as follows: the variance of one phenotype can be decomposed into genetic and environmental factors by making use of the difference between the correlations between MZ twins (who share 100 per cent of their DNA) and DZ twins (who share on average 50 per cent of their segregating genes). Genetic influences can be decomposed into additive genetic factors (A; the sum of allelic effects across all loci) and non-additive genetic factors (D; the effect of genetic dominance and, possibly, epistasis). Similarly, environmental influences can be decomposed into common or shared environmental influences (C; environmental influences that make people from the same family more alike) and non-shared environmental influences (E; environmental influences that make family members less alike) [37, 38].

In the classical twin design, it is not possible to estimate C and D together. The selection of an ACE or an ADE model is made based on the pattern of correlations between MZ and DZ twins. Typically, an ACE model is fitted when the DZ correlation is greater than half of the MZ correlation. On the other hand, an ADE model is fitted when the DZ correlation is less than half of the MZ correlation [38, 39].

Assumptions of the twin design (i.e. equal variances and means for MZ and DZ twins as well as for co-twins) were checked in the saturated models. One univariate model (either ACE or ADE as indicated by the data) was fitted to each of the variables. For all models (univariate and multivariate), nested models (i.e. AE, CE, E) were also fitted to check if one (or two) components could be dropped without a significant decrease in model fit. The fit of the different models and submodels was checked using the likelihood-ratio chi-square test and the Akaike’s information criterion (AIC) [40].

Additionally, we fitted a series of bivariate models to examine the association between chronotype and each of the psychopathology variables. These models allowed us to determine the extent to which the latent variables (i.e. A, C/D, and E) correlate across chronotype and any of the measures of psychopathology. Specifically, these models estimate the etiological correlations (i.e. rA, rC/rD, and rE), which inform us about the degree of overlap between two traits. These range from −1 to 1 where 0 would mean no overlap and 1 or −1 would demonstrate complete overlap. Using the estimates from these models, we then calculated the proportion of each phenotypic correlation explained by A (C or E).

Finally, we fitted a common pathway model (CPM). The CPM posits genetic and environmental factors on a latent variable (here conceptualized as a general psychopathology factor—including chronotype), which then loads onto each variable. Therefore, the genetic and environmental influences on that latent factor can be calculated. In this model, there are also specific paths for the residual genetic and environmental variance of each subscale [39, 41]. This model was used to check the extent to which chronotype can be integrated into a psychopathology factor. A further model (the independent pathways model) was run but is not presented in the main body of this manuscript because it is not as well-placed as the CPM to address the central question of this paper (whether chronotype can be considered a central part of the p-factor). Nonetheless, this model was part of the preregistered statistical plan and was therefore run and added to the Supplementary Material. This model allowed us to test for common genetic and environmental influences directly influencing each of the observed variables, without the need of a higher-order factor [39].

All analyses were run in R version 4.3.2 [42] using the OpenMx package version 2.21.11 [43]. Models were fitted using the direct symmetric approach as this has been proven to have several advantages over the other multivariate models such as the correction of type 1 error rate or parameter bias issues [44]. Most of the variables were in the acceptable range of skewness −2/+2 but paranoia and hallucinations were log-transformed to meet this assumption. Sex was added to the models as a covariate.

#### Polygenic scores

We tested the predictive value of chronotype PGS to predict different aspects of psychopathology. A series of mixed effect regression models were run. We ran one model for each psychopathology trait and phenotypic chronotype (11 models in total) using PGS chronotype as the predictor. These models included family as a random effect added to control for the relatedness of the sample. The analyses were performed using the lme4 package in R [45]. In order to control for multiple testing, we applied a Bonferroni correction (0.05/11 = 0.005). Finally, as an additional analysis, we included a wide range of PGS (for lifestyle variables as well as mental and physical health; Table S1 [all PGS except chronotype]) simultaneously to predict chronotype in a multivariate model including family as a random effect.

### Deviations from the protocol

We performed two sensitivity analyses that were not included in our pre-registration: (a) we removed those who reported using an alarm to wake during free days; (b) we checked whether late chronotype constituted the “risk” group. For this, we examined mean levels of each psychopathology variable across chronotype groups, by splitting the sample into three groups: (1) early group (including the earliest quartile for chronotype), (2) intermediate group (including the second and third quartiles—reflecting the reality that most people fall into this intermediate group), and (3) late group (including the latest quartile).

## Results

### Phenotypic association

Chronotype was significantly associated with all the variables (except callous unemotional traits), and most were above the established 0.1 value (where eveningness was associated with more symptoms of psychopathology). These variables ranged from 0.10 (95% CI, 0.07–0.14) for anxiety and specific psychotic experiences: hallucinations (0.10, 95% CI, 0.07–0.14) to 0.20 (95% CI, 0.17–0.23) for alcohol use. Two further variables were significantly associated with chronotype but the magnitude of the association was very small (eating disorders and autistic traits) and callous unemotional traits did not show a significant association with chronotype (Table 2). Sensitivity analyses removing participants who reported using an alarm clock during free days did not change substantially the significance pattern—and there was a very similar pattern of associations with variations in correlations of <0.02 (Table S2). Further sensitivity analyses examined whether late chronotype was indeed the “risk” group. We found that mean levels of psychopathology were in general similar for the early and intermediate groups, whereas levels of psychopathology were greater for the late group. These analyses support our initial hypothesis that late phenotype is the risk group (Table S3).

**Table 2:** Association Between Chronotype and Psychopathology

Psychopathology measure	Pearson/point-biserial correlation (95% CI)	*P*-value
Symptoms of depression	**0.11 (0.07, 0.14)**	<.001
Symptoms of anxiety	**0.10 (0.07, 0.14)**	<.001
Alcohol use	**0.20 (0.17, 0.23)**	<.001
Callous unemotional traits	0.03 (−0.006, 0.06)	.104
Symptoms of ADHD	**0.18 (0.15, 0.21)**	<.001
Autistic traits	0.06 (0.03, 0.09)	<.001
Symptoms of PTSD	**0.13 (0.10, 0.16)**	<.001
Specific psychotic experiences: paranoia	**0.11 (0.08, 0.14)**	<.001
Specific psychotic experiences: hallucinations	**0.10 (0.07, 0.14)**	<.001
Eating disorder	−0.04 (−0.07, −0.003)^*^	.03

Note: Bold figures represent correlations above 0.1. ADHD: attention deficit hyperactivity disorder; PTSD: post-traumatic stress disorder.

^*^Point-biserial correlation.

### Twin models

All the univariate models for chronotype and associated variables (*r* > 0.1) are presented in Table 3. All intra-pair correlations were higher for MZ twins as compared with DZ twins which is indicative of genetic influence. The best fitting model was always an AE model except for the variable hallucinations and symptoms of ADHD where an ADE model provided the best fit. Genetic factors (A or A + D) explained a substantial proportion of the variance ranging from 31 per cent (paranoia) to 50 per cent (chronotype) (Table 3). Sensitivity analyses which involved removing participants that used an alarm clock during free days showed very similar chronotype correlations for MZ twins (0.53 vs. 0.51) and the same correlation for DZ twins (0.23). This resulted in an almost identical heritability value (51 per cent) (Table S4).

**Table 3:** Univariate Analyses

Variable	**Model**	**Model for comparison**	**A (95% CI)**	**C/D (95% CI)**	**E (95% CI)**	**df**	**−2LL**	**AIC**	**DiffLL**	**Diffdf**	***P***	**rMZ**	**rDZ**
Chronotype												0.51 (0.46, 0.56)	0.23 (0.18, 0.28)
	ADE		0.41 (0.21, 0.61)	0.09 (−0.12, 0.31)	0.50 (0.45, 0.54)	7453	23978.09	23988.09					
	**AE**	ADE	**0.50 (0.45, 0.54)**	*	**0.50 (0.46, 0.55)**	**7454**	**23978.82**	**23986.82**	**0.73**	**1**	**.39**		
	E	AE	*	*	1 (1, 1)	7455	24317.23	24323.23	338.41	1	<.001		
Symptoms of depression												0.41 (0.36, 0.46)	0.22 (0.18, 0.27)
	ACE		0.37 (0.24, 0.50)	0.04 (−0.06, 0.14)	0.59 (0.55, 0.64)	8233	53229.86	53239.86					
	**AE**	**ACE**	**0.42 (0.38, 0.46)**	*	**0.58 (0.54, 0.62)**	**8234**	**53230.49**	**53238.49**	**0.63**	**1**	**.43**		
	CE	ACE	*	0.30 (0.27, 0.33)	0.70 (0.67, 0.73)	8234	53261.30	53269.30	31.44	1	<.001		
	E	AE	*	*	1 (1, 1)	8235	53527.55	53533.55	297.06	1	<.001		
Symptoms of anxiety												0.40 (0.35, 0.45)	0.19 (0.14, 0.24)
	ADE		0.35 (0.15, 0.55)	0.05 (−0.16, 0.27)	0.60 (0.55, 0.64)	7954	53740.59	53750.59					
	**AE**	**ADE**	**0.40 (0.36, 0.45)**	*	**0.60 (0.55, 0.64)**	**7955**	**53740.84**	**53748.84**	**0.25**	**1**	**.62**		
	E	AE	*	*	1 (1, 1)	7956	53988.71	53994.71	247.87	1	<.001		
Alcohol use												0.44 (0.38, 0.49)	0.25 (0.20, 0.30)
	ACE		0.39 (0.25, 0.53)	0.06 (−0.06, 0.17)	0.56 (0.51, 0.61)	7147	42151.89	42161.89					
	**AE**	**ACE**	**0.45 (0.41, 0.50)**	*	**0.55 (0.50, 0.59)**	**7148**	**42152.84**	**42160.84**	**0.95**	**1**	**.33**		
	CE	ACE	*	0.33 (0.29, 0.37)	0.67 (0.63, 0.71)	7148	42180.32	42188.32	28.44	1	<.001		
	E	AE	*	*	1 (1, 1)	7149	42425.82	42431.82	272.98	1	<.001		
Symptoms of ADHD												0.49 (0.44, 0.54)	0.15 (0.09, 0.20)
	**ADE**		**0.08 (−0.13, 0.28)**	**0.42 (0.20, 0.64)**	**0.50 (0.46, 0.55)**	**7568**	**48782.13**	**48792.13**					
	AE	ADE	0.46 (0.42, 0.51)	*	0.54 (0.50, 0.58)	7569	48796.55	48804.55	14.42	1	<.001		
	E	AE	*	*	1 (1, 1)	7570	49078.54	49084.54	282.0	1	<.001		
Symptoms of PTSD												0.42 (0.37, 0.47),	0.22 (0.17, 0.27)
	ACE		0.42 (0.28, 0.56)	0.01 (−0.10, 0.12)	0.57 (0.53, 0.62)	7683	48007.23	48017.23					
	**AE**	**ACE**	**0.43 (0.38, 0.47)**	*	**0.57 (0.53, 0.62)**	**7684**	**48007.25**	**48015.25**	**0.016**	**1**	**.90**		
	CE	ACE	*	0.31 (0.27, 0.34)	0.69 (0.66, 0.73)	7684	48042.48	48050.48	35.25	1	<.001		
	E	AE	*	*	1 (1, 1)	7685	48272.48	48278.48	265.23	1	<.001		
Specific psychotic experiences: paranoia												0.30 (0.25, 0.36)	0.17 (0.12, 0.22)
	ACE		0.26 (0.11, 0.40)	0.05 (−0.07, 0.16)	0.69 (0.65, 0.75)	7565	33415.90	33425.90					
	**AE**	**ACE**	**0.31 (0.27, 0.36)**	*	**0.69 (0.64, 0.73)**	**7566**	**33416.58**	**33424.58**	**0.67**	**1**	**.41**		
	CE	ACE	*	0.23 (0.19, 0.27)	0.77 (0.73, 0.81)	7566	33428.03	33436.03	12.13	1	<.001		
	E	AE	*	*	1 (1, 1)	7567	33564.58	33570.58	148.01	1	<.001		
Specific psychotic experiences: hallucinations												0.31 (0.25, 0.36)	0.11 (0.06, 0.16)
	**ADE**		**0.12 (−0.10, 0.33)**	**0.20 (−0.04, 0.43)**	**0.69 (0.63, 0.74)**	**7563**	**28007.08**	**28017.08**					
	AE	ADE	0.29 (0.24, 0.34)	*	0.71 (0.66, 0.76)	7564	28009.82	28017.82	2.74	1	.10		
	E	AE	*	*	1 (1, 1)	7565	28118.68	28124.68	108.86	1	<.001		

Note: A: additive genetic influences; C: shared environmental influences; D: dominant genetic influences; E: non-shared environmental influences; −2LL: negative 2 log-likelihood; AIC: Akaike’s information criterion; CI: confidence interval; df: degrees of freedom; *p*-value: significance value of the likelihood-ratio chi-square test; rDZ: dizygotic correlations; rMZ: monozygotic correlations. Bold text indicates the best fitting model.

Multivariate models showed significant genetic correlations between chronotype and all included measures of psychopathology ranging from 0.09 (with paranoia) to 0.28 (with symptoms of ADHD). We also found significant non-shared environmental correlations with chronotype although of lower magnitude (ranging from 0.06 [for hallucinations] to 0.14 [for alcohol]). The phenotypic variance was explained by both genetic and environmental factors (Table 4 and Figures S1–S7).

**Table 4:** Multivariate Twin AE Models

Psychopathology measure	rPH	rG	rE	Proportion of correlation due to	
				A	E
Symptoms of depression	0.15 (0.12, 0.17)	0.16 (0.09, 0.24)	0.13 (0.08, 0.18)	0.51 (0.30, 0.71)	0.49 (0.29, 0.70)
Symptoms of anxiety	0.14 (0.11, 0.16)	0.22 (0.14, 0.29)	0.07 (0.02, 0.13)	0.71 (0.49, 0.93)	0.29 (0.07, 0.51)
Alcohol use	0.19 (0.17, 0.21)	0.24 (0.17, 0.31)	0.14 (0.09, 0.20)	0.60 (0.43, 0.76)	0.40 (0.24, 0.57)
Symptoms of ADHD	0.18 (0.15, 0.20)	0.28 (0.21, 0.35)	0.09 (0.03, 0.14)	0.75 (0.58, 0.91)	0.25 (0.09, 0.42)
Symptoms of PTDS	0.15 (0.13, 0.17)	0.22 (0.14, 0.29)	0.09 (0.04, 0.15)	0.66 (0.46, 0.85)	0.34 (0.15, 0.54)
Specific psychotic experiences: paranoia	0.11 (0.09, 0.13)	0.09 (0.01, 0.18)	0.12 (0.07, 0.18)	0.33 (0.01, 0.61)	0.67 (0.39, 0.99)
Specific psychotic experiences: hallucinations	0.09 (0.06, 0.11)	0.14 (0.05, 0.24)	0.06 (0.01, 0.11)	0.61 (0.22, 0.98)	0.39 (0.02, 0.78)

Note: the best fitting model for symptoms of ADHD was an ADE model but for the sake of clarity we present here the AE model including all the genetic influences in the A component. ADHD: attention deficit hyperactivity disorder; PTSD: post-traumatic stress disorder.

**Figure 1:**
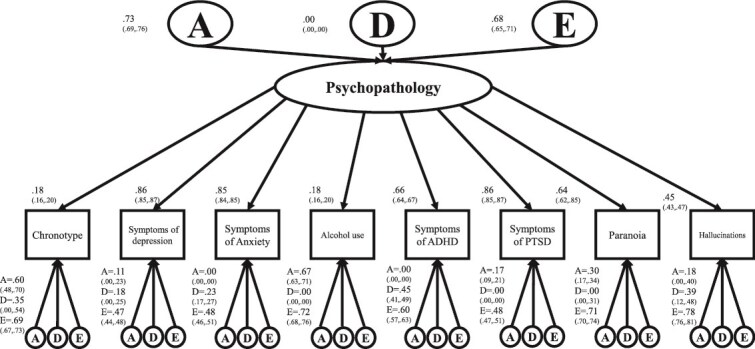
Common pathway model.

We also fitted a CPM to test how chronotype is integrated into the latent factor—general psychopathology. This model is depicted in Figure 1. The best fit was provided by an ADE model (AIC_ADE_ = 309960.2 vs. AIC_AE_ = 309961.9; *p* = .02). The latent factor was almost equally explained by genetic (0.73^2^ = 54 per cent) and environmental factors (0.68^2^ = 0.46 per cent). Factor loadings were in general high except for chronotype (0.18) and use of alcohol (0.18). This result suggests that genetic/environmental influences included in the psychopathology p-factor do not explain variance for chronotype substantially. Specifically, the variance of chronotype explained by this latent factor was around 2 per cent for genetic factors ((0.73^*^0.18)^2^ = 0.017) and 1.5 per cent for non-shared environmental factors ((0.68^*^0.18)^2^ = 0.015), with the rest attributable to specific genetic/environmental factors (96.5 per cent). As expected, results from the independent pathway model (Fig. S8) showed a similar pattern where just 12 per cent of the variance was shared with the other phenotypes (0.07^2^ + 0.31^2^ + 0.15^2^ = 0.12). Also similarly, the best fit was provided by an ADE model (AIC_ADE_ = 309549.2 vs. AIC_AE_ = 309769.5; *p* < .001).

### Polygenic scores

In Table 5, the predictive value of PGS calculated for chronotype predicting different aspects of psychopathology is presented (see Table S5 for additional analyses whereby PGS for the different aspects of psychopathology are predictors of chronotype). As expected, PGS for chronotype significantly predicted our chronotype measure and explained 1.79 per cent of the variance. This PGS also significantly predicted symptoms of ADHD (*p* = .003) although explained <0.2 per cent of the variance. As we ran 11 tests (one for each measure), we corrected for multiple testing (0.05/11 = 0.005). After Bonferroni correction, only the model predicting ADHD symptoms from chronotype PGS remained significant. Finally, as an exploratory analysis, we examined whether several lifestyle, physical, and mental health PGS predicted chronotype. Most of the PGS did not significantly predict chronotype (Table S5). Only the PSGs for schizophrenia, autism spectrum disorder, and ever having smoked were significantly associated to chronotype. These PGS each explained around 0.3 per cent of the variance for chronotype (e.g. *R*^2^ of the model (1.49 per cent) − *R*^2^ of the model without PGS for schizophrenia (1.20 per cent = 0.29 per cent change).

**Table 5:** Regression Models Examining the Predictive Value of Chronotype PGS for Psychopathology and Phenotypic Chronotype

Variable	*R* ^2^ (%)	*P*-value
Chronotype	1.79	<.001
Symptoms of depression	0.069	.069
Symptoms of anxiety	0.036	.196
Alcohol use	0.142	.014
Callous unemotional trait	0.023	.315
Symptoms of ADHD	0.197	.003
Autistic traits	0.043	.165
Symptoms of PTDS	0.076	.065
Specific psychotic experiences: paranoia	0.06	.104
Specific psychotic experiences: hallucinations	0.001	.833
Eating disorder	0.031	.238

Note: As we did 11 tests (one for each measure), we corrected for multiple testing (0.05/11 = 0.005) so alcohol did not remain significant after the multiple test correction (although ADHD remained significant). ADHD: attention deficit hyperactivity disorder; PTSD: post-traumatic stress disorder.

## Discussion

In this study, we explored the associations between chronotype and different measures of psychopathology. Furthermore, these associations were tested using different methods. First, we examined the phenotypic associations. Second, different twin models were applied to disentangle the role of genetic and environmental influences on this association. Finally, we tested the predictive value of a chronotype PGS for a wide range of psychopathology traits (and examined PGSs for different traits as predictors of chronotype). Our results highlight a modest, albeit significant, association between chronotype and psychopathology. We also found some genetic and environmental overlap and that PGS for chronotype predict some measures of psychopathology (e.g. symptoms of ADHD).

The associations between chronotype and measures of psychopathology were in the expected direction and of a similar magnitude to previous studies. For example, in a study of college students, the associations between chronotype and a composite measure of depression, anxiety, and stress was in the 0.1–0.2 range—similar to associations with related phenotypes reported in our study [46]. Similarly, with regard to depression, a meta-analysis showed that eveningness is significantly associated with depression although the magnitude of this association is small [4]. A similar picture can be found for alcohol and symptoms of ADHD which show small associations with chronotype [47, 48].

Regarding the genetic and environmental influences on chronotype, we found that this phenotype is equally influenced by genetic and environmental factors. This is in line with findings from previous studies [12, 49, 50]. When it comes to genetic and environmental influences on the associations with psychopathology, the comparison with other studies is more difficult since there is a scarcity of studies addressing this issue. However, there are some exceptions, for example, in a study using a sample of male twin pairs, it was found that being more of an evening type was associated with greater symptoms of depression with a genetic correlation of .21 and an environmental correlation of .10 (non-significant) [12]. We found significant genetic and environmental correlations of a similar magnitude. Another study found that genetic influences accounted for a substantial proportion of the covariance between diurnal preference and externalizing behaviors (80 per cent) [16]. Although not the same trait, in this study, we found that the association between chronotype and symptoms of ADHD was mainly explained by genetic factors (75 per cent; proportion of the phenotypic correlation due to A).

It has been proposed previously that chronotype could be a transdiagnostic correlate of psychopathology and that often the evening chronotype precedes psychopathology [19]. Furthermore, variation in circadian factors could influence the response to certain medications [51]. Our study aimed to test to what extent chronotype could be integrated into the p-factor [17]. Our results show that genetic and environmental influences common to this p-factor when it comes to chronotype are very low (around 3–4 per cent). This does not align with our hypothesis. We found in our multivariate models that there is a significant genetic and environmental overlap but when a CPM was fitted, the majority of the variance of chronotype was explained by specific factors to this trait rather than those common to psychopathology. Overall, this suggests that while there is a small association between chronotype and psychopathology, our data do not support the idea that it is part of the central “p-factor.”

Finally, we also tested the predictive value of PGS calculated for chronotype to predict measures of psychopathology. We found that this PGS was significant for symptoms of ADHD although the predictive value was low. Interestingly, and in line with this finding, one of the highest genetic correlations was found between symptoms of ADHD and chronotype. This might suggest that from a genetic perspective, of all the different aspects of psychopathology examined in this study, ADHD may be of most interest in relation to chronotype [52]. Nonetheless, these results need to be considered within the wider context that the predictive value of PGS in general are still limited and the genetic correlations that we found were of a moderate magnitude. Another interesting result is that PGS for schizophrenia, autism spectrum disorder, and ever having smoked significantly predict chronotype suggesting that there is a significant genetic overlap between chronotype and these phenotypes (as also shown by twin models).

The causal mechanisms underlying the association between chronotype and psychopathology are still largely unknown. Some mechanisms have been proposed such as structural brain or neuroedocrinology differences, social factors, or psychological factors among others [53]. However, further research is needed to study this association from a longitudinal perspective in genetically informative designs to comprehensively study this association.

This study has several strengths such as the use of a representative large twin sample and the inclusion of a wide range of measures of psychopathology. Nevertheless, our results must also be interpreted in light of limitations. First, we used a reduced version of the MCTQ to avoid over-burdening participants. Nonetheless, this version has been shown to be reliable [26]. Future studies should also include other measures of psychopathology to validate these results. This study focused on twins aged 26 and may not apply to other developmental stages such as childhood, middle-, or older-adulthood. This is particularly noteworthy given developmental changes in chronotype [54]. It may be that the links between chronotype and psychopathology manifest in different ways at different stages of the life course. For example, if a late chronotype led to greater social jetlag, sleep deprivation, and lifestyle disruption, it may be that the associations with mental health increase over time. Further research should examine whether associations between chronotype and the p-factor become stronger with age. Another limitation is the use of different versions of LDpred used to compute polygenic scores. This could have affected the predictive performance of scores. Nevertheless, previous research suggests that such differences are unlikely to be substantial [55]. Finally, the cross-sectional nature of our study does not allow us to draw conclusions about the direction of effects between variables or to establish causal associations. Further research using longitudinal designs is therefore needed.

## Conclusions

This study investigated for the first time the association between chronotype and psychopathology comprehensively in a genetically informative design. Our results show that there is a significant association between these phenotypes and that there is also a significant genetic and environmental overlap although the magnitude is low/moderate. Our results also showed that PGS for chronotype predict some measures of psychopathology (symptoms of ADHD). However, taken together, our results do not support the idea that chronotype should be considered part of the p-factor and further research is needed to elucidate the complex associations between chronotype and psychopathology.

## Supplementary Material

SupplementaryFiles_Final_zsaf231

## Data Availability

By application.
