# Supplementary material for: Chronotype and psychopathology: insights from a genetically informative design
Source: Sleep. 2025 Sep 10;49(3):zsaf231. doi: 10.1093/sleep/zsaf231 (PMC13017916; doi:10.1093/sleep/zsaf231)

**Chronotype and psychopathology: insights from a genetically informative design**

Juan J Madrid-Valero^1,2^; Juan R Ordoñana^1,2^; Thalia C Eley^3,4^, Alice M Gregory^5^

^1^Department of Human Anatomy and Psychobiology, University of Murcia, Murcia, Spain.

^2^Murcia Institute for Biomedical Research (IMIB-Arrixaca), Murcia, Spain

^3^Social, Genetic and Developmental Psychiatry Centre, Institute of Psychiatry, Psychology and Neuroscience, King's College London, London, United Kingdom.

^4^National Institute for Health and Care Research (NIHR) Maudsley Biomedical Research Centre, South London and Maudsley NHS Foundation Trust, London, UK

^5^Department of Psychology, Royal Holloway, University of London, London, UK

**Corresponding authors:**

Juan J Madrid-Valero – Department of Human Anatomy and Psychobiology. University of Murcia. Campus de Espinardo 30100, Murcia (Spain) Telephone: +34 868887831/ Email: [juanjose.madrid1@um.es](mailto:juanjose.madrid1@um.es); Orcid: 0000-0002-3450-1159

Alice M Gregory – Department of Psychology, Wolfson Building, Royal Holloway, Egham, Surrey, TW20 0EX

Email: [Alice.gregory@rhul.ac.uk](mailto:Alice.gregory@rhul.ac.uk); ORCID: 0000-0003-2222-7823

**Table of contents**

-Supplementary Table 1: List of Polygenic scores

-Supplementary Table 2: Sensitivity analysis for the association between chronotype and psychopathology

-Supplementary Table 3 Descriptive statistics across chronotype groups

-Supplementary Table 4: Univariate model for chronotype removing participants that used an alarm clock during free days

-Supplementary Table 5: PGS predicting chronotype

-Supplementary Figure 1: Bivariate model chronotype and symptoms of Depression (AE best fitting model)

-Supplementary Figure 2: Bivariate model chronotype and symptoms of anxiety

(AE best fitting model)

-Supplementary Figure 3: Bivariate model chronotype and alcohol use (AE best fitting model)

-Supplementary Figure 4: Bivariate model chronotype and symptoms of ADHD (AE best fitting model)

-Supplementary Figure 5: Bivariate model chronotype and symptoms of PTSD (AE best fitting model)

-Supplementary Figure 6: Bivariate model chronotype and Specific Psychotic Experiences: Paranoia (AE best fitting model)

-Supplementary Figure 7: Bivariate model chronotype and Specific Psychotic Experiences: Hallucinations (AE best fitting model)

-Supplementary Figure 8: ADE Independent Pathway Model

**Supplementary Tables**

Supplementary Table 1: List of Polygenic scores

| PGS | Software | Publication |
| --- | --- | --- |
| Chronotype | LDpred1 | UKbioBank |
| Depression | LDpred2 | Howard et al., (2019) |
| Anxiety | LDpred2 | Purves et al., (2020) |
| Alcohol dependence | LDpred2 | Walters et al., (2018) |
| Cannabis use disorder | LDpred2 | Johnson et al., (2020) |
| Ever Smoked | LDpred1 | UKbioBank |
| Risk taking | LDpred1 | UKbioBank |
| ADHD | LDpred2 | Demontis et al., (2023) |
| Aggression | LDpred2 | Ip et al., (2021) |
| Autism spectrum disorder | LDpred2 | Grove et al., (2019) |
| PTSD | LDpred2 | Nievergelt., (2019) |
| Obsessive-compulsive disorder | LDpred1 | International Obsessive Compulsive Disorder Foundation Genetics Collaborative (IOCDF-GC) and OCD Collaborative Genetics Association Studies (OCGAS)(2018) |
| Anorexia nervosa | LDpred2 | Watson et al., (2019) |
| Bipolar disorder | LDpred2 | Mullins et al., (2021) |
| Panic disorder | LDpred2 | Forstner et al., (2021) |
| Schizophrenia | LDpred2 | Trubetskoy et al., (2022) |
| Time spent at the computer | LDpred1 | UKbioBank |
| Time spent watching TV | LDpred1 | UKbioBank |
| Overall health | LDpred1 | UKbioBank |

ADHD: attention deficit hyperactivity disorder; PDSD: post-traumatic stress disorder

Supplementary Table 2: Sensitivity analysis for the association between chronotype and psychopathology

| Psychopathology measure | Pearson/ Point-biserial correlation (95% CI) | P-Value |
| --- | --- | --- |
| Symptoms of depression | 0.10 (0.06,0.13) | <0.001 |
| Symptoms of anxiety | 0.09 (0.05,0.13) | <0.001 |
| Alcohol use | 0.21 (0.18,0.25) | <0.001 |
| Callous Unemotional Trait | 0.03 (-0.01.0.07) | 0.09 |
| Symptoms of ADHD | 0.18 (0.14,0.22) | <0.001 |
| Autistic traits | 0.05 (0.01,0.08) | 0.014 |
| Symptoms of PTDS | 0.11 (0.07,0.15) | <0.001 |
| Specific Psychotic Experiences: paranoia | 0.10 (0.07,0.14) | <0.001 |
| Specific Psychotic Experiences: hallucinations | 0.09 (0.05,0.12) | <0.001 |
| Eating disorder | -0.02 (-0.06,0.02)* | 0.30 |

ADHD: attention deficit hyperactivity disorder; PTSD: post-traumatic stress disorder

*Point-biserial correlation

Supplementary Table 3 Descriptive statistics across chronotype groups

|  | **Early group**  **N=1941** | **Intermediate group**  **N=3880** | **Late group**  **N=1940** |
| --- | --- | --- | --- |
| Mean symptoms of depression (SD) | 6.44 (6.12) | 6.1 (5.92) | 8.16 (6.54) |
| Mean symptoms of anxiety (SD) | 7.35 (7.24) | 6.98 (6.77) | 8.99 (7.68) |
| Mean alcohol use (SD) | 5.28 (4.26) | 6.46 (4.34) | 7.94 (5.57) |
| Mean symptoms of ADHD (SD) | 5.94 (5.44) | 6.00 (5.85) | 8.42 (6.95) |
| Mean symptoms of PTSD (SD) | 5.16 (5.51) | 4.92 (5.28) | 6.76 (6.06) |
| Mean specific psychotic experiences: paranoia (SD) | 7.06 (11.39) | 6.16 (10.31) | 8.86 (12.41) |
| Mean specific psychotic experiences: hallucinations (SD) | 1.15 (3.53) | 0.91 (2.89) | 1.66 (4.33) |

ADHD: attention deficit hyperactivity disorder; PTSD: post-traumatic stress disorder; SD: standard deviation.

Note: groups were calculated using quartiles. The early group included the first quartile, the intermediate group quartiles two and three and the late group included the forth quartile.

Supplementary Table 4: Univariate model for chronotype removing participants that used an alarm clock during free days

|  | **Model** | **Model for**  **comparison** | **A (95% CI)** | **C/D (95% CI)** | **E (95% CI)** | **df** | **-2LL** | **AIC** | **DiffLL** | **Diffdf** | **P** | **rMZ** | **rDZ** |
| --- | --- | --- | --- | --- | --- | --- | --- | --- | --- | --- | --- | --- | --- |
| Chronotype |  |  |  |  |  |  |  |  |  |  |  | 0.53 (0.47,0.58) | 0.23 (0.17,0.29) |
|  | ADE |  | 0.40 (0.12,0.63) | 0.14 (-0.12,0.42) | 0.47 (0.42,0.53) | 5865 | 18886.71 | 18896.71 |  |  |  |  |  |
|  | **AE** | ADE | **0.51 (0.46,0.56)** | ***** | **0.49 (0.43,0.54)** | **5866** | **18887.89** | **18895.89** | **1.17** | **1** | **0.28** |  |  |
|  | E | AE | * | * | 1 (1,1) | 5867 | 19107.00 | 19113.00 | 219.11 | 1 | <0.001 |  |  |

Supplementary Table 5: PGS predicting chronotype

| PGS | R^2^ of the model after removing each PGS (%) | P-value |
| --- | --- | --- |
| Depression | 1.49% | 0.700 |
| Anxiety | 1.49% | 0.818 |
| Alcohol dependence | 1.48% | 0.524 |
| Cannabis use disorder | 1.46% | 0.252 |
| Ever Smoked | 1.19% | <0.001 |
| Risk taking | 1.46% | 0.229 |
| ADHD | 1.47% | 0.343 |
| Aggression | 1.45% | 0.181 |
| Autism spectrum disorder | 1.21% | <0.001 |
| Obsessive-compulsive disorder | 1.46% | 0.232 |
| Anorexia nervosa | 1.48% | 0.537 |
| Bipolar disorder | 1.47% | 0.377 |
| PTSD | 1.41% | 0.068 |
| Overall health | 1.43% | 0.116 |
| Panic disorder | 1.49% | 0.695 |
| Schizophrenia | 1.20% | <0.001 |
| Time spent at the computer | 1.45% | 0.210 |
| Time spent watching TV | 1.48% | 0.549 |

R^2^ full model: 1.49%

ADHD: attention deficit hyperactivity disorder; PDSD: post-traumatic stress disorder

Demontis, D., Walters, G. B., Athanasiadis, G., Walters, R., Therrien, K., Nielsen, T. T., . . . Consortium, i.-B. (2023). Genome-wide analyses of ADHD identify 27 risk loci, refine the genetic architecture and implicate several cognitive domains. *Nat Genet, 55*(2), 198-208. doi:10.1038/s41588-022-01285-8

Forstner, A. J., Awasthi, S., Wolf, C., Maron, E., Erhardt, A., Czamara, D., . . . Schumacher, J. (2021). Genome-wide association study of panic disorder reveals genetic overlap with neuroticism and depression. *Mol Psychiatry, 26*(8), 4179-4190. doi:10.1038/s41380-019-0590-2

Grove, J., Ripke, S., Als, T. D., Mattheisen, M., Walters, R. K., Won, H., . . . Team, a. R. (2019). Identification of common genetic risk variants for autism spectrum disorder. *Nat Genet, 51*(3), 431-444. doi:10.1038/s41588-019-0344-8

Howard, D. M., Adams, M. J., Clarke, T. K., Hafferty, J. D., Gibson, J., Shirali, M., . . . Consortium, M. D. D. W. G. o. t. P. G. (2019). Genome-wide meta-analysis of depression identifies 102 independent variants and highlights the importance of the prefrontal brain regions. *Nat Neurosci, 22*(3), 343-352. doi:10.1038/s41593-018-0326-7

International Obsessive Compulsive Disorder Foundation Genetics Collaborative (IOCDF-GC) and OCD Collaborative Genetics Association Studies (OCGAS). (2018). Revealing the complex genetic architecture of obsessive-compulsive disorder using meta-analysis. *Mol Psychiatry, 23*(5), 1181-1188. doi:10.1038/mp.2017.154

Ip, H. F., van der Laan, C. M., Krapohl, E. M. L., Brikell, I., Sánchez-Mora, C., Nolte, I. M., . . . Boomsma, D. I. (2021). Genetic association study of childhood aggression across raters, instruments, and age. *Transl Psychiatry, 11*(1), 413. doi:10.1038/s41398-021-01480-x

Johnson, E. C., Demontis, D., Thorgeirsson, T. E., Walters, R. K., Polimanti, R., Hatoum, A. S., . . . Workgroup, P. G. C. S. U. D. (2020). A large-scale genome-wide association study meta-analysis of cannabis use disorder. *Lancet Psychiatry, 7*(12), 1032-1045. doi:10.1016/S2215-0366(20)30339-4

Mullins, N., Forstner, A. J., O'Connell, K. S., Coombes, B., Coleman, J. R. I., Qiao, Z., . . . Psychiatry, H. A.-I. (2021). Genome-wide association study of more than 40,000 bipolar disorder cases provides new insights into the underlying biology. *Nat Genet, 53*(6), 817-829. doi:10.1038/s41588-021-00857-4

Nievergelt, C. M., Maihofer, A. X., Klengel, T., Atkinson, E. G., Chen, C. Y., Choi, K. W., . . . Koenen, K. C. (2019). International meta-analysis of PTSD genome-wide association studies identifies sex- and ancestry-specific genetic risk loci. *Nat Commun, 10*(1), 4558. doi:10.1038/s41467-019-12576-w

Purves, K. L., Coleman, J. R. I., Meier, S. M., Rayner, C., Davis, K. A. S., Cheesman, R., . . . Eley, T. C. (2020). A major role for common genetic variation in anxiety disorders. *Mol Psychiatry, 25*(12), 3292-3303. doi:10.1038/s41380-019-0559-1

Trubetskoy, V., Pardiñas, A. F., Qi, T., Panagiotaropoulou, G., Awasthi, S., Bigdeli, T. B., . . . Consortium, S. W. G. o. t. P. G. (2022). Mapping genomic loci implicates genes and synaptic biology in schizophrenia. *Nature, 604*(7906), 502-508. doi:10.1038/s41586-022-04434-5

Walters, R. K., Polimanti, R., Johnson, E. C., McClintick, J. N., Adams, M. J., Adkins, A. E., . . . Team, a. R. (2018). Transancestral GWAS of alcohol dependence reveals common genetic underpinnings with psychiatric disorders. *Nat Neurosci, 21*(12), 1656-1669. doi:10.1038/s41593-018-0275-1

Watson, H. J., Yilmaz, Z., Thornton, L. M., Hübel, C., Coleman, J. R. I., Gaspar, H. A., . . . Consortium, E. D. W. G. o. t. P. G. (2019). Genome-wide association study identifies eight risk loci and implicates metabo-psychiatric origins for anorexia nervosa. *Nat Genet, 51*(8), 1207-1214. doi:10.1038/s41588-019-0439-2

**Supplementary Figures**

Supplementary Figure 1: Bivariate model chronotype and symptoms of Depression (AE best fitting model)


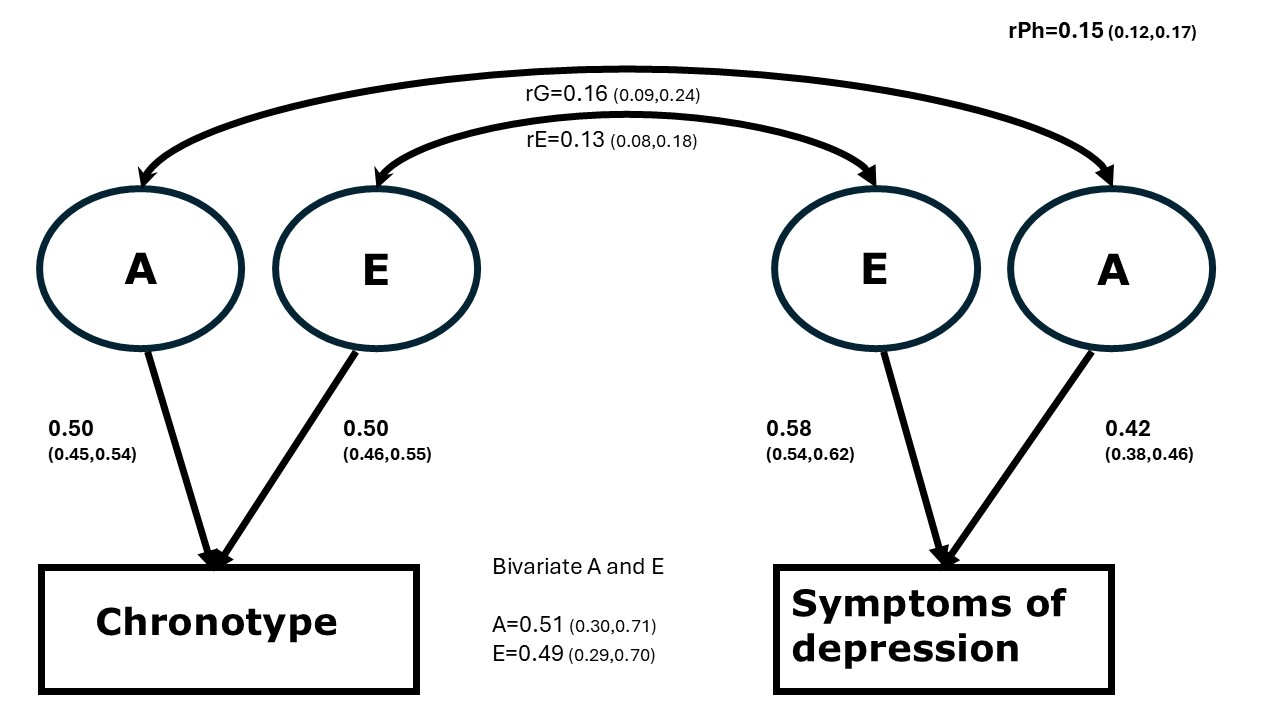


Supplementary Figure 2: Bivariate model chronotype and symptoms of anxiety (AE best fitting model)


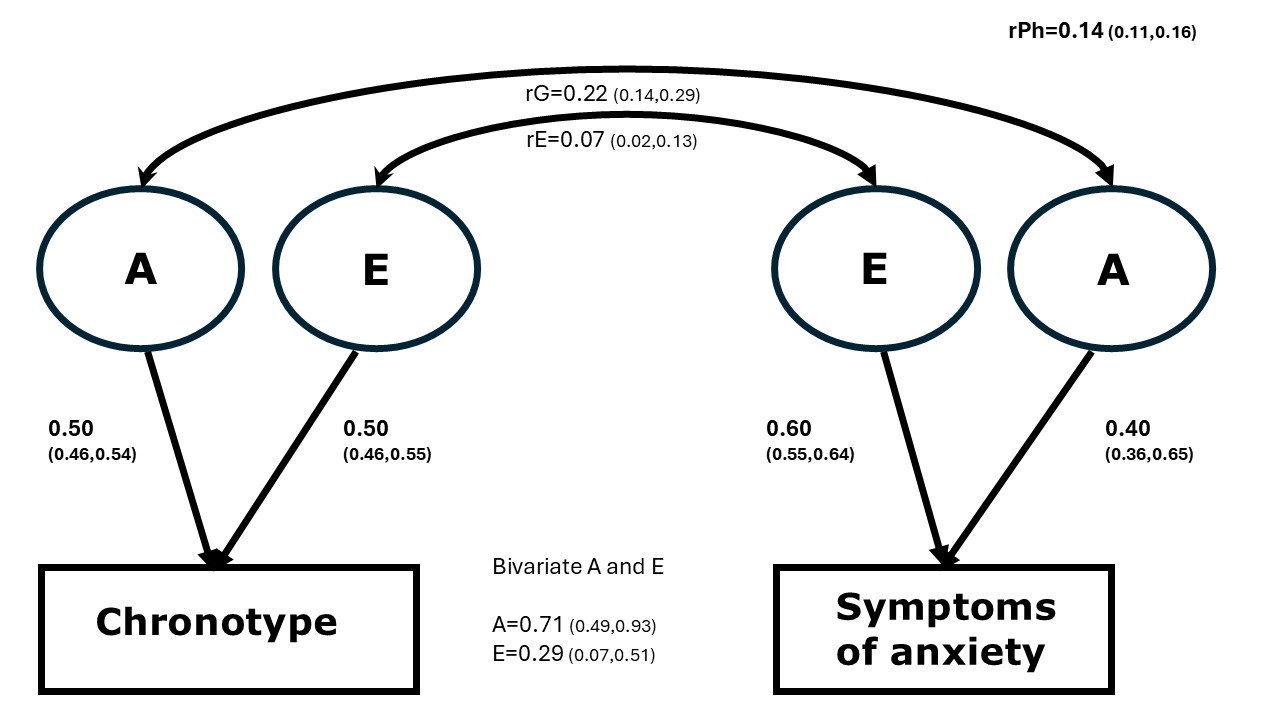


Supplementary Figure 3: Bivariate model chronotype and alcohol use (AE best fitting model)


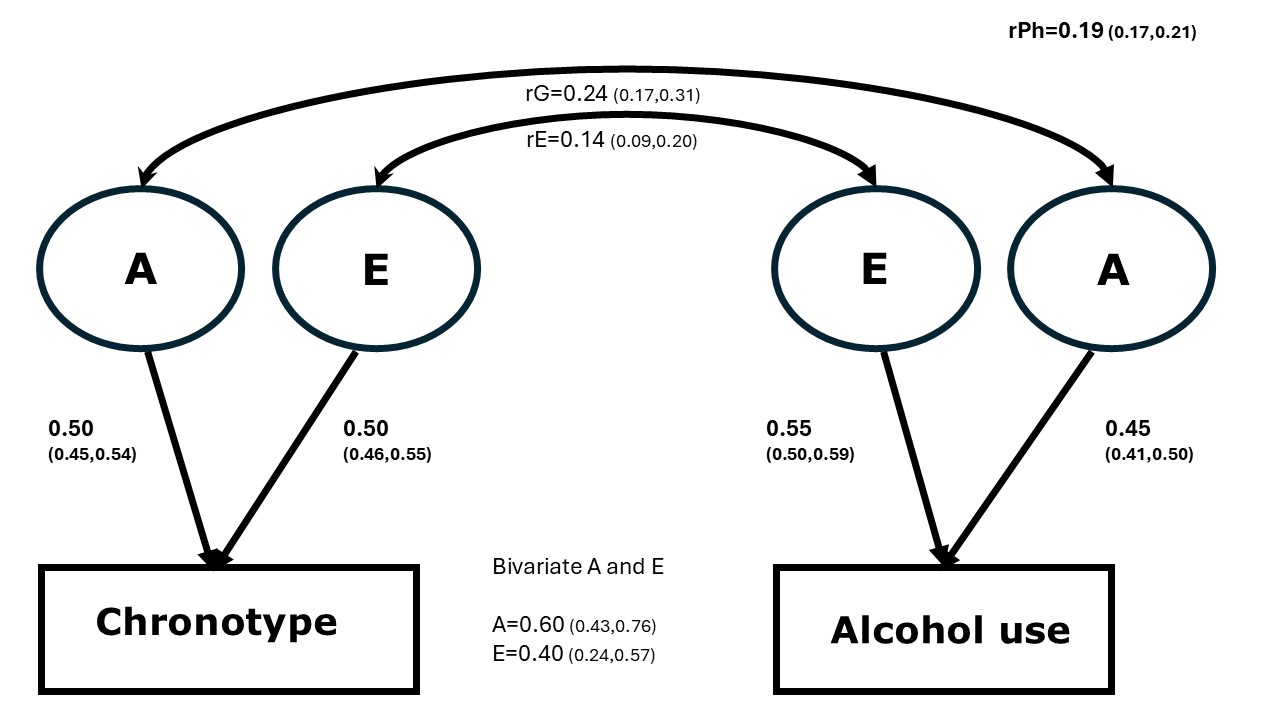


Supplementary Figure 4: Bivariate model chronotype and symptoms of ADHD (AE best fitting model)


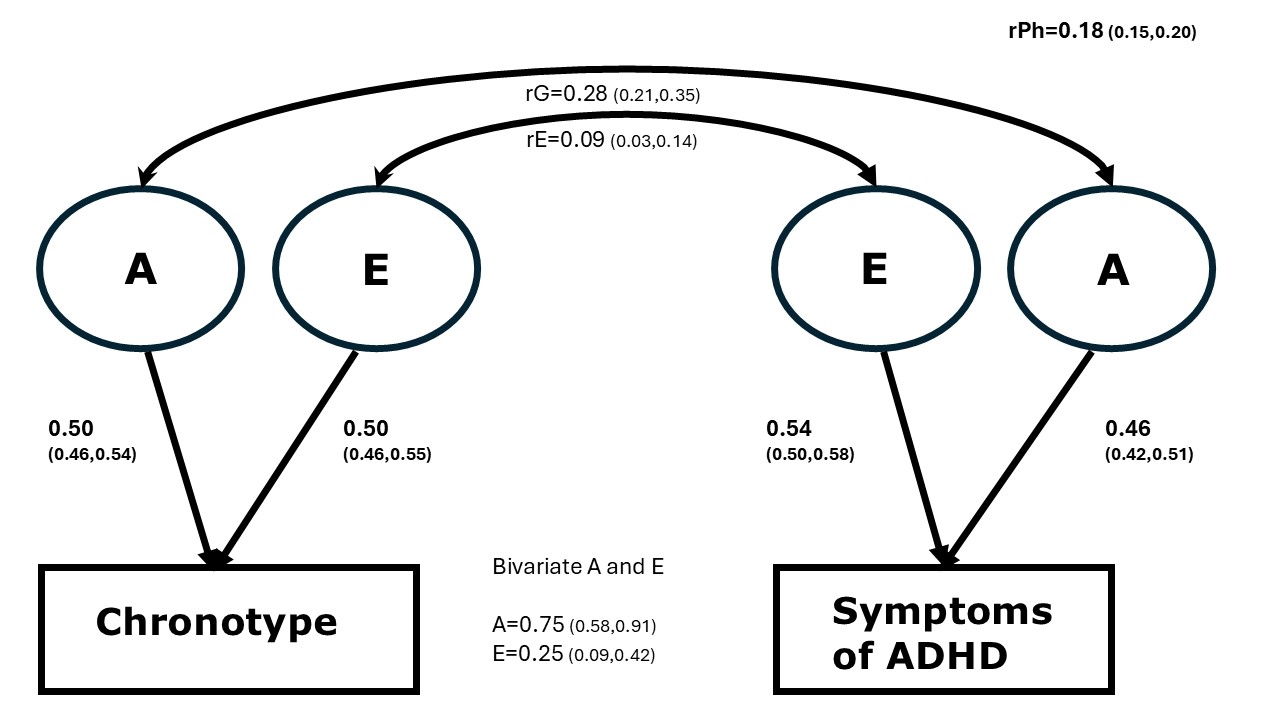


Supplementary Figure 5: Bivariate model chronotype and symptoms of PTSD (AE best fitting model)


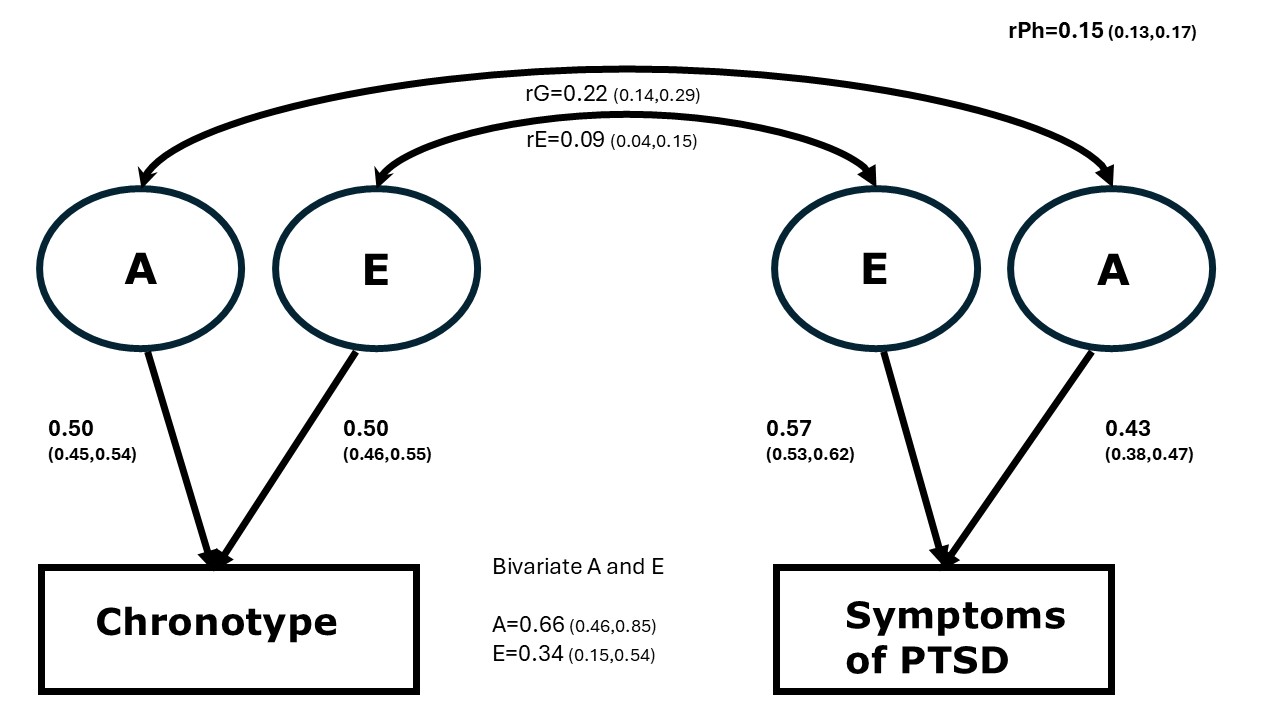


Supplementary Figure 6: Bivariate model chronotype and Specific Psychotic Experiences: Paranoia (AE best fitting model)


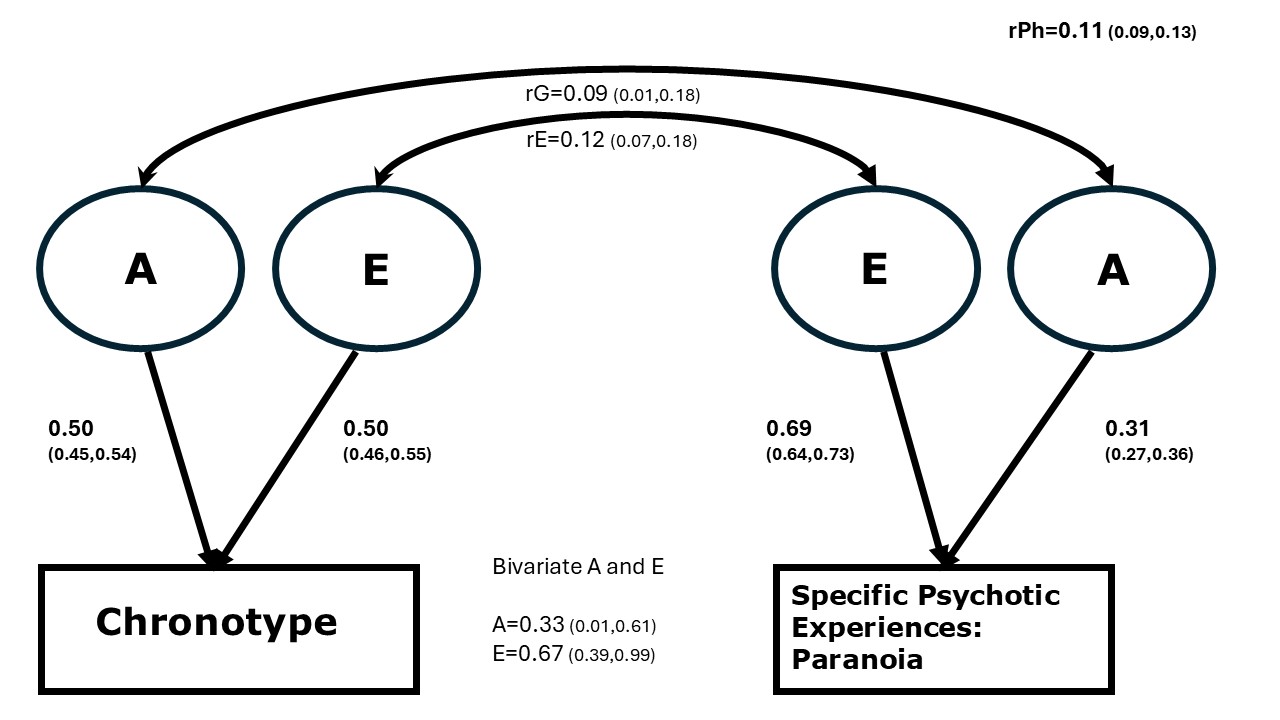


Supplementary Figure 7: Bivariate model chronotype and Specific Psychotic Experiences: Hallucinations (AE best fitting model)


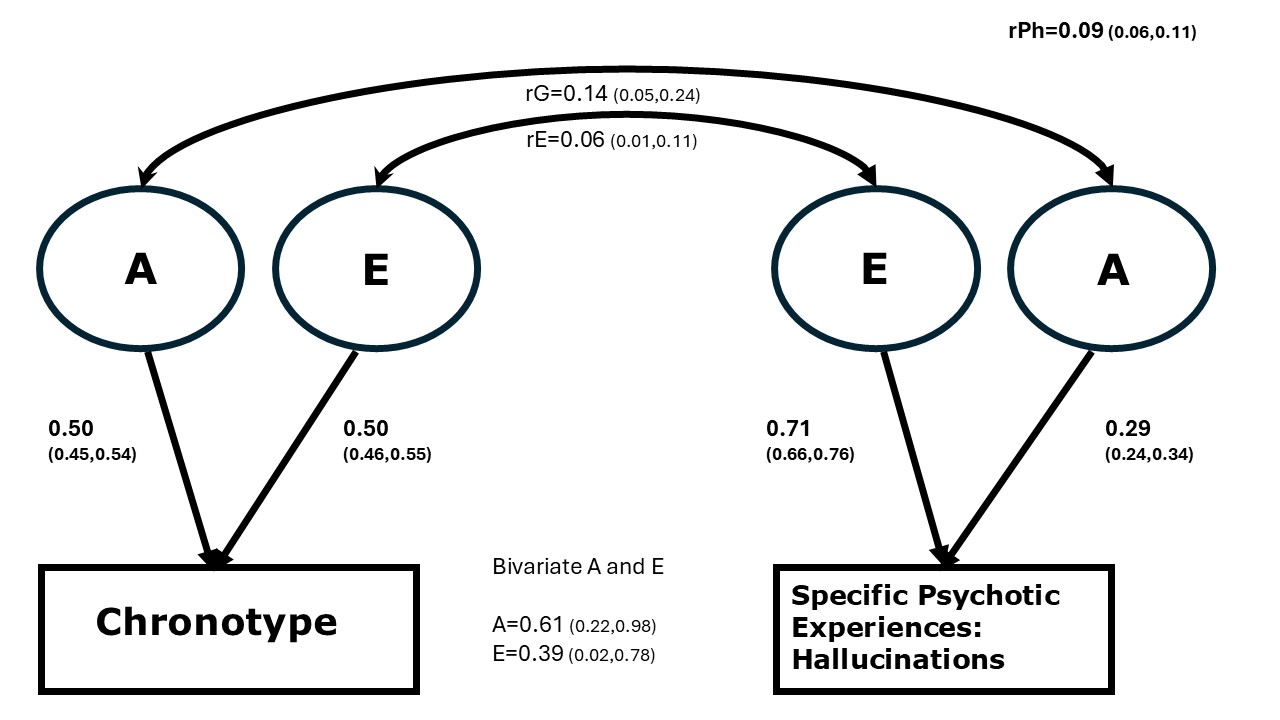


Supplementary Figure 8: ADE Independent Pathway Model


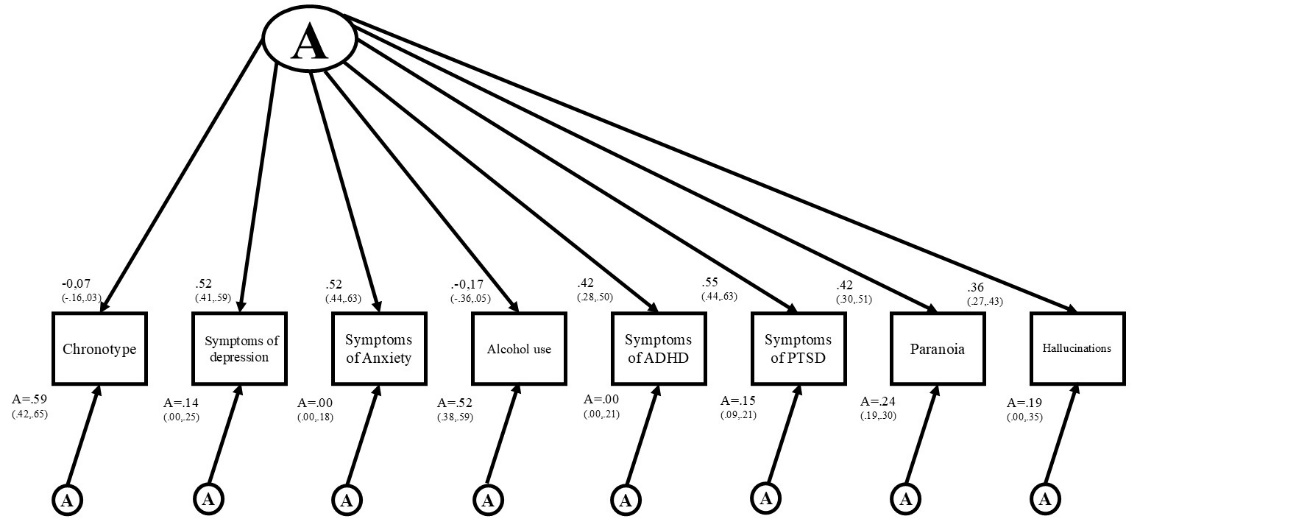


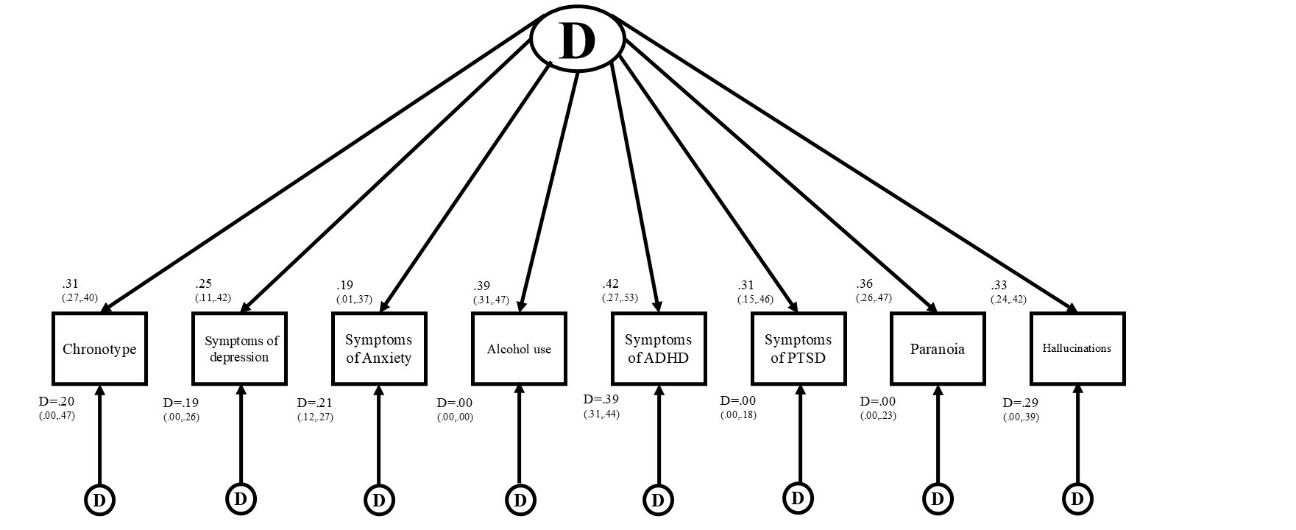


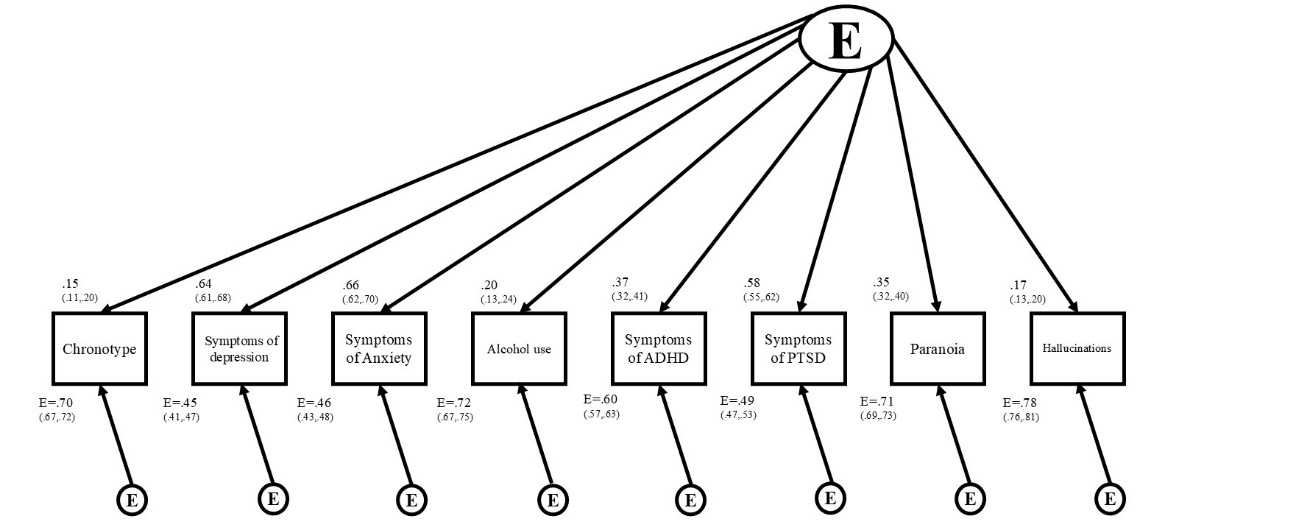

Supplement: SupplementaryFiles_Final_zsaf231 [file supplementaryfiles_final_zsaf231.docx]
